# Docimologic analysis of child psychiatry examination

**DOI:** 10.1192/j.eurpsy.2025.2373

**Published:** 2025-08-26

**Authors:** S. Bourgou, N. Boujelbene, N. Ben Salah, G. Amira

**Affiliations:** 1 Faculty of Medicine of Tunis, Tunis; 2 Child psychiatry Department, Mongi Slim Hospital, La Marsa, Tunisia

## Abstract

**Introduction:**

Docimology is the science that assesses the quality of tests and items, based on different indices and coefficients.

**Objectives:**

The purpose of our study was to perform a docimological analysis, based on docimological indices, of the items and exams of Child psychiatry of psychiatry certificates porposed for the students of the Third Year of the Second Cycle of Medical Studies in the Faculty of Medicine of Tunis.

**Methods:**

We carried out a retrospective and descriptive study. We have included the scores of main sessions’ psychiatry certificate exam of six academic years (2016-2017 to 2021- 2022). We did not include the scores of this certificate obtained at the control sessions during the period of our study. We carried out a global docimological analysis of the psychiatry exam, of the child psychiatry exam and its items.

**Results:**

We included a total of 2780 exam scripts spread over 12 main sessions. We found an annual pass rate of 96.7% in the psychiatry certificate and 85.3% in the discipline of child psychiatry. The study of the internal homogeneity of the psychiatric tests showed that the Alpha index of Cronbach varied between 0.64 and 0.82 with an average index of 0.74 which corresponds to an internal homogeneity at least acceptable. We found that the maximum scores obtained in the discipline of child psychiatry varied from 16.5 to 19.75 out of 20. The average rate of students, who passed the psychiatry certificate test without passing the discipline child psychiatry, was 11.9%. The questions were easy in 51.7% (62 questions) ans have at least good discrimination in 31.7%. We found also that 25 questions (65,7%) were “ideal”.

**Conclusions:**

Child psychiatry examination in the Faculty of Medicine of Tunis meets globally the docimologic recommendations. This is the first step to build up a bank of items regularly enriched with “ideal” questions with metric qualities known in advance.

**Disclosure of Interest:**

None Declared

